# Gut dysbacteriosis induces expression differences in the adult head transcriptome of *Spodoptera frugiperda* in a sex-specific manner

**DOI:** 10.1186/s12866-023-03089-0

**Published:** 2023-12-07

**Authors:** Junrui-Fu, Zixia Rong, Ximei Huang, Junhan Wang, Xiaoyan Long, Qili Feng, Huimin Deng

**Affiliations:** 1 Guangdong Key Laboratory of Insect Developmental Biology and Applied Technology, Guangzhou Key Laboratory of Insect Development Regulation and Application Research, Institute of Insect Science and Technology & School of Life Sciences, South China Normal University, Guangzhou, 510631 China; 2 Guangdong Laboratory for Lingnan Modern Agriculture, Guangzhou, 510631 China; 3 Guangmeiyuan R&D Center, Guangdong Provincial Key Laboratory of Insect Developmental Biology and Applied Technology, South China Normal University, Meizhou, 514779 China

**Keywords:** *Spodoptera frugiperda*, Gut microbiota, Head transcriptome, Immunity and neurodevelopment

## Abstract

Mounting evidence indicates that the gut microbiota influences the neurodevelopment and behavior of insects through the gut-brain axis. However, it is currently unclear whether the gut microbiota affect the head profiles and immune pathway in pests. Here, we find that gut bacteria is essential for the immune and neural development of adult *Spodoptera frugiperda*, which is an extremely destructive agricultural pest worldwide. 16 S rRNA sequencing analysis showed that antibiotics exposure significantly disturbed the composition and diversity of gut bacteria. Further transcriptomic analysis revealed that the adult head transcripts were greatly affected by gut dysbacteriosis, and differently expression genes critical for brain and neural development including *A4galt*, *Tret1*, *nsun4*, *Galt*, *Mitofilin*, *SLC2A3*, *snk*, *GABRB3*, *Oamb* and *SLC6A1* were substantially repressed. Interestingly, the dysbacteriosis caused sex-specific differences in immune response. The mRNA levels of *pll* (serine/threonine protein kinase Pelle), *PGRP* (peptidoglycan-sensing receptor), *CECA* (cecropin A) and *CECB* (cecropin B) involved in Toll and Imd signaling pathway were drastically decreased in treated male adults’ heads but not in female adults; however, genes of *HIVEP2*, *ZNF131*, *inducible zinc finger protein 1-like* and *zinc finger protein 99-like* encoding zinc-finger antiviral protein (ZAP) involved in the interferon (IFNα/β) pathway were significantly inhibited in treated female adults’ heads. Collectively, these results demonstrate that gut microbiota may regulate head transcription and impact the *S. frugiperda* adults’ heads through the immune pathway in a sex-specific manner. Our finding highlights the relationship between the gut microbiota and head immune systems of *S. frugiperda* adults, which is an astonishing similarity with the discoveries of other animals. Therefore, this is the basis for further research to understand the interactions between hosts and microorganisms via the gut-brain axis in *S. frugiperda* and other insects.

**Supplementary Information:**

The online version contains supplementary material available at 10.1186/s12866-023-03089-0.

## Background

The bidirectional interactions of the nervous and immune systems have been widely demonstrated in vertebrates [1, 2]. Some compounds released by immune cells cause changes in neuronal activities [3]. In some cases, it can lead to changes in behavioral coordination. On the other hand, the nervous system changes immune function through the endocrine system [4]. Therefore, the immune and nervous systems are involved in the crosstalk related to function and homeostasis. It is the main function of the immune system, serving as a sensory organ and sending signals to the central nervous system, monitoring immune status and microbial challenges [5]. More studies have suggested that interactions between the nervous and immune systems are no longer unique to vertebrates. The physiological behavioral phenomena in insects are similar to those in vertebrates [6]. The stimulation of the immune response can cause insect anorexia, changes in behavior and reproduction, and a decrease in learning abilities [7, 8].

Recently, many studies have demonstrated that gut microbes have underestimated potential to contribute to brain development and function. The metabolites short-chain fatty acids (SCFAs) and others derived from the gut microbiome regulate the behavior of immune cells throughout the body, including those in the brain [9]. Neuroactive compounds produced by gut bacteria influence brain function and behavior [10], leading to nervous disorders [11, 12]. Two-way regulation of the gut microbiota-brain axis in mammalian models has revealed the contribution of microbiota to the occurrence of neurodegenerative diseases [13, 14] and emotional regulation, including anxiety and depression [15]. Recent studies have also shown a link between gut dysbiosis and social barriers, such as autism spectrum disorder (ASD) [16] and schizophrenia [17]. However, studies on the connections between the gut microbiota and brain have mainly focused on vertebrate model organisms, such as mice and rats [18]. As one of the most diverse species in the world, insects possess more diverse gut microorganisms affecting development, life span, reproduction and immune responses [19]. The insect brain controls the chemical sense, physiological state and behavioral ability. The gut microbiota has shown profound effects on odorant profiles, olfactory behavior [20], and neurophysiological development [21] in insects. For example, the lower termite *Reticulitermes speratus* can easily recognize and attack invaders that are colonized by foreign gut bacteria, promoting unfamiliar scents [22]. Gut dysbacteriosis interferes with the hive behaviors of nurse bees, affecting neurotransmitter concentrations, protein levels in the brain and circulating metabolic profiles [23]. Compared with fruit flies fed traditional gut microbiota, axenic *Drosophila* have worse learning and memory in aversive phototactic assays [24]. To date, the comprehensive understanding of these interactions is still limited due to only a few model organisms being used, resulting in the inability to truly understand the proximate mechanisms involved in this phenomenon.

In Lepidoptera, *Spodoptera frugiperda* (Lepidoptera: Noctuidae) is a typical agricultural pest that feeds on more than 300 host plant species and has strong adaptability, flight capabilities and reproductive capability [25, 26]. Due to the influence of the environment, development stage and host plants, *S. frugiperda* has diverse gut microbial species. The phyla Firmicutes and Bacteroidetes dominate across *S. frugiperda* life stages, and the genera *Enterococcus*, *Enterobacter*, *Acinetobacter*, *Bacillus* and *Klebsiella* are significantly enriched and more frequent in different samples, especially *Enterococcus*, which is a core species [27–31]. There are only a few reports on the specific regulation and molecular mechanism of the gut microbiota on *S. frugiperda* physiological functions. Antibiotic exposure could affect energy and metabolic homeostasis in *S. frugiperda* [32], and the larval food intake and body weight of *S. frugiperda* decreased, and the larval stage was prolonged due to changes in the gut microbiota [33]. The field-collected larvae showed greater potential for insecticides than those from laboratory-selected resistant strains, which is an important reason for *S. frugiperda* resistance to various pesticides in the field [34]. Therefore, further studies are necessary to explore the functional roles of the gut microbiota in *S. frugiperda.*

To fully appreciate the role of bacterial symbionts in the evolution and brain behavior of *S. frugiperda*, the potential impact of gut microbiota on the head transcriptome was investigated in this study. First, *S. frugiperda* with gut dysbacteriosis was established, and RNA-seq results proved that gut bacteria could impact the head transcriptional program, especially immune pathways, and the immune response to gut microbiome changes occurred in a sex-specific manner. Finally, the expression levels of genes related to the immune response in the brain were determined by qRT‒PCR. This information will help to further investigate the roles of gut microbes in the behavior of *S. frugiperda* and may be a feasible strategy for future insect biological management by adjusting insect behavioral traits.

## Materials and methods

### Insect rearing and antibiotic treatment

The population of *S. frugiperda* originally collected from corn fields in Dali city (Yunnan Province, China) was established and maintained in the laboratory. After hatching from eggs, *S. frugiperda* larvae were fed an artificial diet as previously described [35]. All larvae and adults were reared under the conditions of temperature 26 ± 1 °C, relative humidity 65%±5%, and a photoperiod of 14:10 h (light: dark). The emerged adults were supplied with 10% honey solution. For the same batch of egg masses, parts of the egg masses were raised routinely, while others were collected 48 h after laying and dechorionated for 4 min in 4% formaldehyde solution, immersed in 8% sodium hypochlorite solution (containing 4% sodium hydroxide) for 3 min, and rinsed twice with sterile water [36]. The eggs were then transferred to beef-protein medium and allowed to develop. After confirmation of free bacterial contamination, the newly hatched larvae were transferred to a sterile artificial diet that was mixed with a combination of antibiotics (gentamycin, penicillin, streptomycin, ciprofloxacin hydrochloride, rifampicin and vancomycin, 100 µg/mL each). To maintain sterile conditions, all culture tubes and tools underwent autoclave sterilization, and all manipulations were performed in a biosafety cabinet. The pupae that emerged from the treated larvae were transferred to sterile tissue culture bottles. Adults were fed a 10% sterile honey solution mixed with a combination of antibiotics. Eggs were adhered to sterile spawning papers in bottles. Larvae and adults of the next generation continued to be reared through the same process. Then, the second generation of treated adults and untreated adults were collected for subsequent experiments.

### Extraction of gut samples and 16 S rRNA sequencing

Conventional female adult (C-female) and male adult (C-male) gut samples, and treated female adult (T-female) and male adult (T-male) gut samples (3 days old) were collected for microbiome analysis. On a super clean bench, all procedures were performed with sterile scalpel tweezers on ice. Gut samples were rinsed in 75% ethanol for 30 s and rinsed again in sterile 0.1mol PBS (pH 7.2). A total of 15 moths from each group were used for three biological replicates. Using HiPure Soil DNA Kits (Magen, Guangzhou, China), bacterial DNA was extracted according to the manufacturer’s protocols. The V3-V4 region of the 16S rRNA gene was amplified using the primers 341F (5’-CCTACGGGNGGCWGCAG-3’) and 806R (5’-GGACTACHVGGGTATCTAAT-3’) [37]. Amplicons were extracted from 2% agarose gels and purified using an AxyPrep DNA Gel Extraction Kit (Axygen Biosciences, Union City, CA, U.S.) according to the manufacturer’s instructions and quantified using an ABI StepOnePlus Real-Time PCR System (Life Technologies, Foster City, USA).

The purified amplicons were pooled in equimolar amounts and paired-end sequenced (PE250) on an Illumina NovaSeq 6000 platform. DNA sequences for this experiment are available at the NCBI Sequence Read Archive (SRA) database. Raw 16 S rRNA fastq files were quality filtered according to the standard protocols described previously [38]. OTUs were clustered using a cutoff of 97% similarity with the UPARSE (version 9.2.64) pipeline [39] and clustered into organisms through the naïve Bayesian model using the RDP classifier (version 2.2) [40] based on the SILVA database (version 132) [41] and UNITE database (version 8.0) [42] with a confidence threshold value of 0.8. The stacked bar plot of the community composition at the phylum or genus level was visualized in R project ggplot2 package (version 2.2.1) [43]. Chao1 and Shannon index were all measured based on OTUs at the species level with QIIME (version 1.9.1) [44]. Principal coordinate analysis (PCoA) of weighted UniFrac distances was used to compare bacterial diversity between groups with the Vegan package of R program (version 2.5.3) [45]. Linear discriminant analysis coupled with effect size (LEfSe) analysis (LDA score > 2 and *P* < 0.05) was used to identify distinguishing genus of each group [46].

Kyoto Encyclopedia of Genes and Genomes (KEGG) pathway analysis was performed using Tax4Fun (version 1.0) [47]. Functional differences between groups were calculated by Welch’s *t* test and Kruskal‒Wallis H test or Tukey’s HSD test in the Vegan package of R program (version 2.5.3) [48].

### RNA extraction and transcriptome sequencing

Virgin adult head samples were collected at 72 h after emergence for transcriptome analysis, and all procedures were performed with sterile tweezers and petri dishes on ice. Moreover, corresponding brain and antenna samples were also extracted for subsequent qRT-PCR analysis. Samples were rinsed in 75% ethanol for 30 s and rinsed again in sterile 0.1 mol PBS (pH 7.2), followed by tissue dissection. Three biological replicates (total 15 moths per group) of frozen samples were ground in liquid nitrogen. Using the TRIzol reagent kit (Invitrogen, Carlsbad, CA, USA), total RNA was isolated according to the manufacturer’s protocol. RNA quality was assessed by RNase-free agarose gel electrophoresis in an Agilent 2100 Bioanalyzer (Agilent Technologies, Palo Alto, CA, USA).

### RNA sequencing analysis

After total RNA was extracted, eukaryotic mRNA was enriched by Oligo (dT) beads. Then the enriched mRNA was fragmented into short fragments using fragmentation buffer and reversely transcribed into cDNA by using NEBNext Ultra RNA Library Prep Kit for Illumina (NEB #7530, New England Biolabs, Ipswich, MA, USA).The purified double-stranded cDNA fragments were end repaired, a base added, and ligated to Illumina sequencing adapters. The ligation reaction was purified with the AMPure XP Beads(1.0X).And polymerase chain reaction (PCR) amplified.The resulting cDNA library was sequenced using Illumina Novaseq6000 by Gene Denovo Biotechnology Co. (Guangzhou, China). Reads were further filtered by fastp [49] (version 0.18.0) to get high quality clean reads and short reads alignment tool Bowtie2 [50] (version 2.2.8) was used for mapping reads to ribosome. Paired-end clean reads were further used in assembly and mapped to the reference assembly genome ZJU_Sfru_1.0 (Assembly accession: GCF_011064685.1) using HISAT2. 2.4 [51]. The mapped reads of each sample were assembled using StringTie v1.3.1 [52] in a reference-based approach. For each transcription region, a FPKM (fragment per kilobase of ranscript per million mapped reads) value was calculated to quantify its expression abundance and variations using RSEM [53] software. RNAs differential expression analysis was performed by DESeq2 [54] software between two different groups. Analysis of differential expression between two different groups was performed using DESeq2 software [55]. Transcripts with a false discovery rate (FDR) below 0.05 and absolute fold change ≥ 2 were considered differentially expressed.

Gene Ontology (GO) and KEGG pathway enrichment analyses were conducted to analyze the biological function. All DEGs were mapped to GO terms in the Gene Ontology database (http://www.geneontology.org/), gene numbers were calculated for every term, significantly enriched GO terms in DEGs comparing to the genome background were defined by hypergeometric test. Significantly enriched GO terms and pathways of the genes were defined by a hypergeometric test and a threshold of FDR less than 0.05 [56]. In addition, gene set enrichment analysis (GSEA) and MSigDB software programs [57] were used to identify specific GO terms/pathways and show significant differences between the two groups. Enrichment scores and *p* values were calculated with the default parameters.

### Quantitative real-time PCR

Quantitative real-time PCR (qRT-PCR) was performed using SYBR Green I Master Mix in a standard 96-well block to measure gene expression levels. The relative expression level of genes was determined by the 2^−ΔΔCt^ method with the gene *GAPDH* (glyceraldehyde-3-phosphate dehydrogenase, GenBank ID: 118,271,716) as an internal reference. Three biological replicates were performed in the same sample. The primers used in this study are listed in Table S1.

### Statistical analyses

Statistical analysis between the control and treated groups was conducted using Student’s *t* test for unpaired comparisons. When *p* < 0.05, the difference was considered statistically significant. All statistics were completed using GraphPad 5.0. All data are displayed as the mean ± standard error (SEM). Welch’s *t* test in R Project was used to analyze the sequencing data of gut microbiota.

## Results

### Effect of antibiotic treatment on gut microbiota composition and function

To test whether the gut microbiota impacts the head transcriptome of *S. frugiperda* adults, the amplicons of 16 S rRNA genes isolated from the gut microbiota of 12 adult samples were sequenced first. A total of 1,578,994 raw reads with an average length of 472.58 bp were obtained. The reads with sequence similarity greater than 97% at the species level were clustered into 2656 OTUs. Among them, OTUs were distributed in four groups, as follows: C-female (308 OTUs), C-male (532 OTUs), T-female (903 OTUs), and T-female (913 OTUs) (Table S2).

**Fig. 1:**
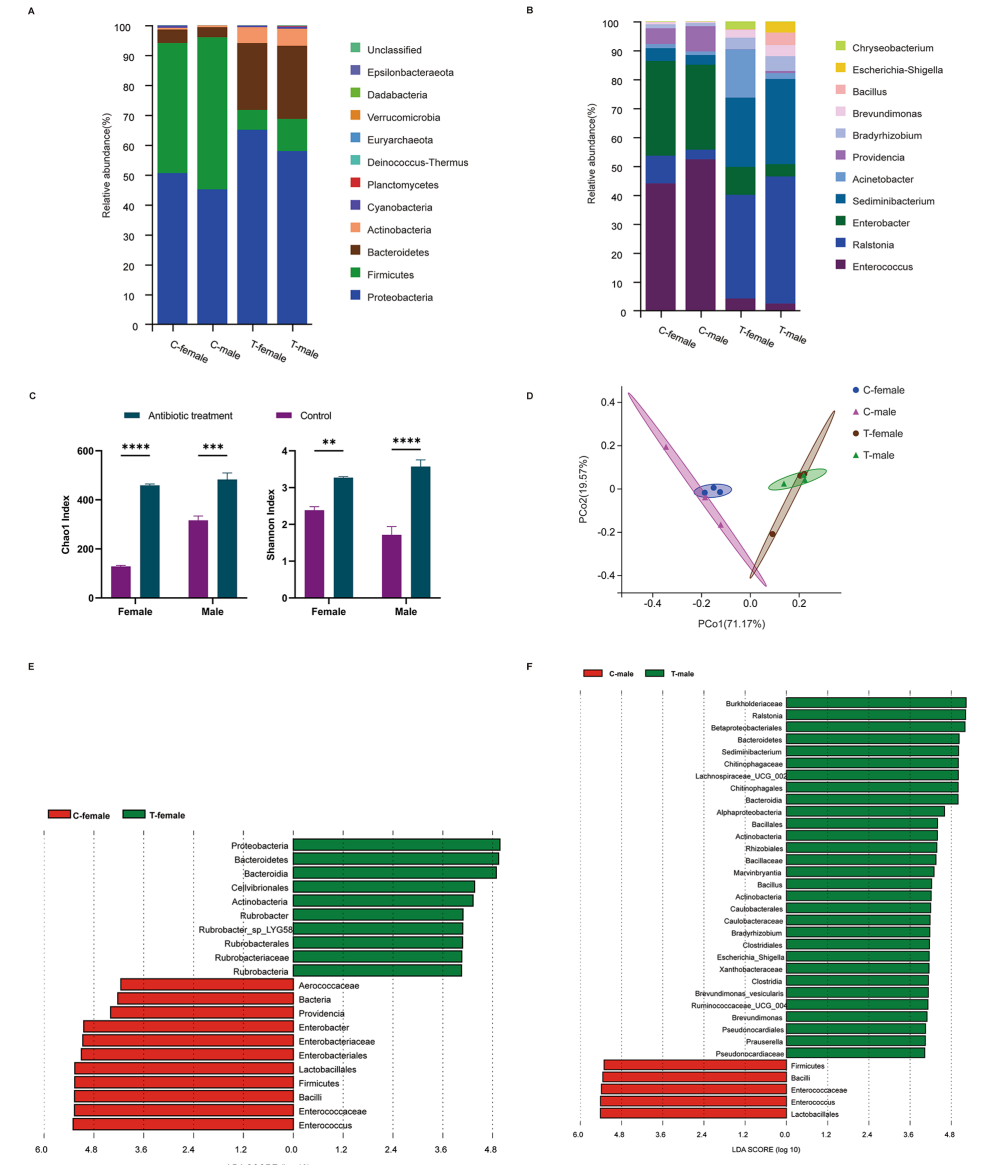
Effects of antibiotic treatment on adult gut microbiota components and diversity in *S. frugiperda*. (**A-B**) Relative abundance of gut microbiota at the phylum level (**A**) and genus level (**B**) in the guts of adults developed from larvae reared on a control diet without antibiotics and an antibiotic-containing diet. (**C**) Alpha diversity is displayed by the Chao1 index and Shannon index. The significance of the differences between the control and treatment was statistically analyzed by using the Kruskal‒Wallis test at *p* < 0.0001 (****), *p* < 0.001 (***), and *p* < 0.01 (**). (**D**) Beta diversity was displayed by principal coordinate analysis (PCoA) based on the weighted UniFrac method at the OTU level. C-female and C-male represent the control females and males, respectively. T-female and T-male represent the antibiotic-treated females and males, respectively. (**E-F**) Linear discriminant analysis (LDA) effect size (LEfSe) plot of taxonomic biomarkers identified in the adult gut microbiome of *S. frugiperda* females and males (LDA > 4, *p* < 0.05)

Composition analysis of the adult gut microbiome demonstrated that the microbial species in the gut were similar between virgin female and male adults (Fig. 1A-B). Proteobacteria (female, 50.52%; male, 45.07%) and Firmicutes (female, 43.62%; male, 51.00%) (average value across all of the samples) were the dominant phyla in the control adult guts, followed by Bacteroidetes and Actinobacteria (Fig. 1A). In antibiotic-treated adults, a decrease in Firmicutes (female, 5.73%; male, 8.90%) and an increase in Bacteroidetes (female, 22.59%; male, 25.46%) were observed (Fig. 1A). Similarly, the relative abundances of *Enterococcus* (female, 42.48%; male, 52.53%) and *Enterobacter* (female, 31.48%; male, 28.97%) in the control groups were dramatically greater than those *in the treated groups* (female, 3.62%; male, 2.13%; female, 8.74%; male, 3.67%) (Fig. 1B). In addition, *Ralstonia* (female, 29.82%; male, 37.80%) and *Sediminibacterium* (female, 19.90%; male, 25.30%) were shown to be the most obviously increased genera (Fig. 1B). These results suggest that antibiotic treatment results in dysbacteriosis of the adult gut. Furthermore, the antibiotic-treated adults showed a significant increase in bacterial α-diversity, as evidenced by the Chao1 and Shannon indices (Fig. 1C). The weighted UniFrac PCoA showed two independent clusters with the bacterial composition between conventional and antibiotic-treated groups, profound along the PCO1 axis (reaching 71.17% of overall variation), while no significant difference was found between the female and male adults (Fig. 1D). To identify the dominant bacterial species in these two groups, linear discriminant effect size (LEfSe) analysis was performed, which showed 21 differential bacterial taxa in females and 31 differential bacterial taxa in males (Fig. 1E-F). Firmicutes and *Enterococcus* had higher relative abundances in both the untreated females and males. However, the most differentially abundant bacterial taxa in antibiotic-treated female adults were Proteobacteria and *Rubrobacter*, while the key bacterial taxa Bacteroidetes and *Ralstonia* were more abundant in the antibiotic-treated male groups (Fig. 1E-F). In brief, these results suggest that antibiotic exposure contributes to gut microbiota dysbiosis.

**Fig. 2:**
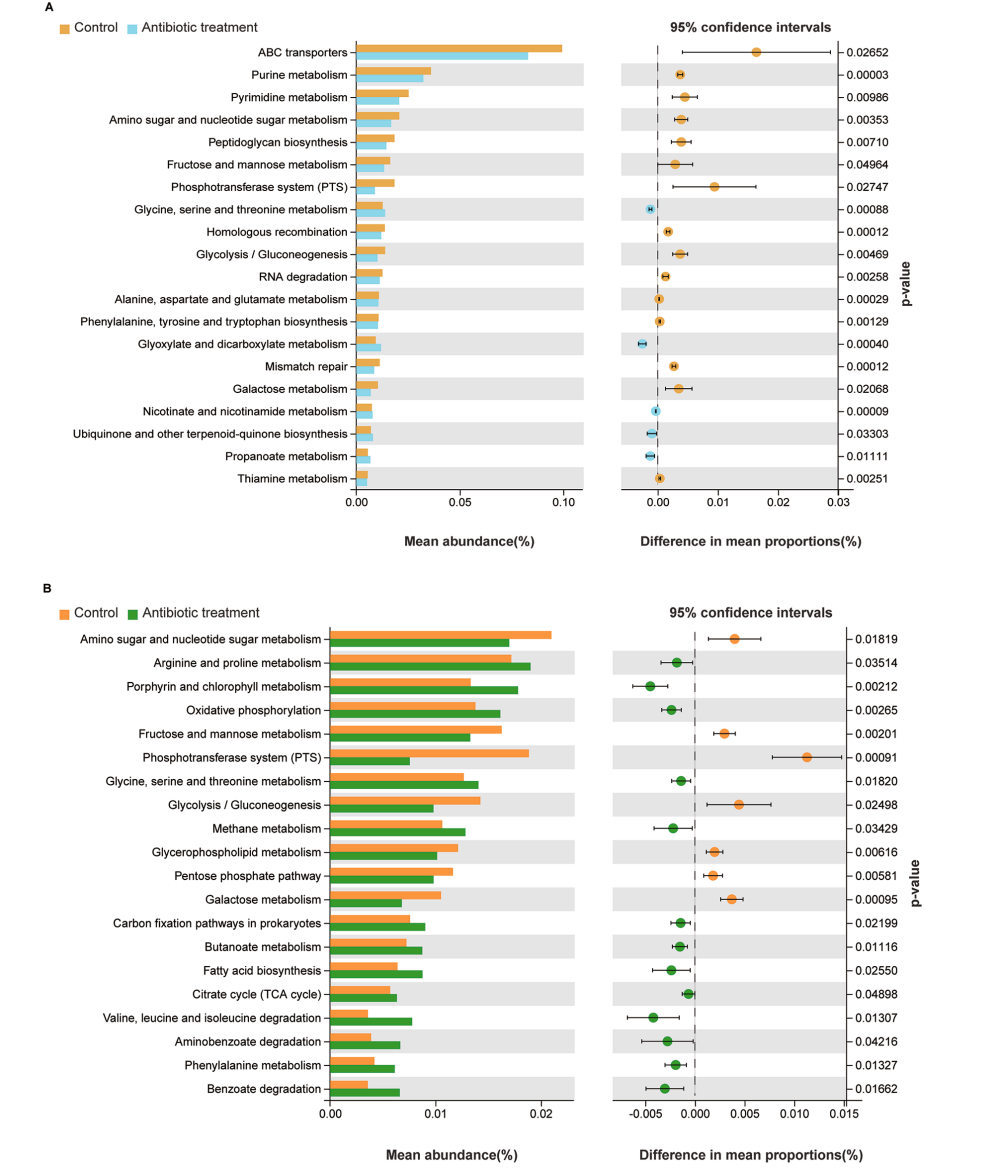
Inferred functions of gut bacterial communities. All of the predicted KEGG metabolic pathways are shown at the third hierarchical level and grouped by major functional categories (Welch´s *t* test, *p < 0.05). (**A**) Female adults. (**B**) Male adults

Furthermore, the Tax4Fun program was used to predict gut microbiota functions. As shown in Fig. 2A, the top 20 pathways at the third level of KEGG, such as ABC transporters, purine metabolism, pyrimidine metabolism, amino sugar and nucleotide sugar metabolism, peptidoglycan biosynthesis, fructose and mannose metabolism, and phosphotransferase system (PTS), were enriched differently between the treated female and the control female adults (Fig. 2A). The top 20 pathways such as amino sugar and nucleotide sugar metabolism, arginine and proline metabolism, porphyrin and chlorophyll metabolism, oxidative phosphorylation, fructose and mannose metabolism, phosphotransferase system (PTS), glycine, serine and threonine metabolism were significantly affected in male adults after antibiotic exposure (Fig. 2B). In particular, the most related KEGG pathways were metabolism which were less enriched in all treated groups (Fig. 2A-B). These data reveals that gut microbiota affects host through their biological functions regulated by the genes of gut microbiota, causing female and male adults to have different metabolic responses.

### Sex differences in the head transcriptome between female and male adults

**Fig. 3:**
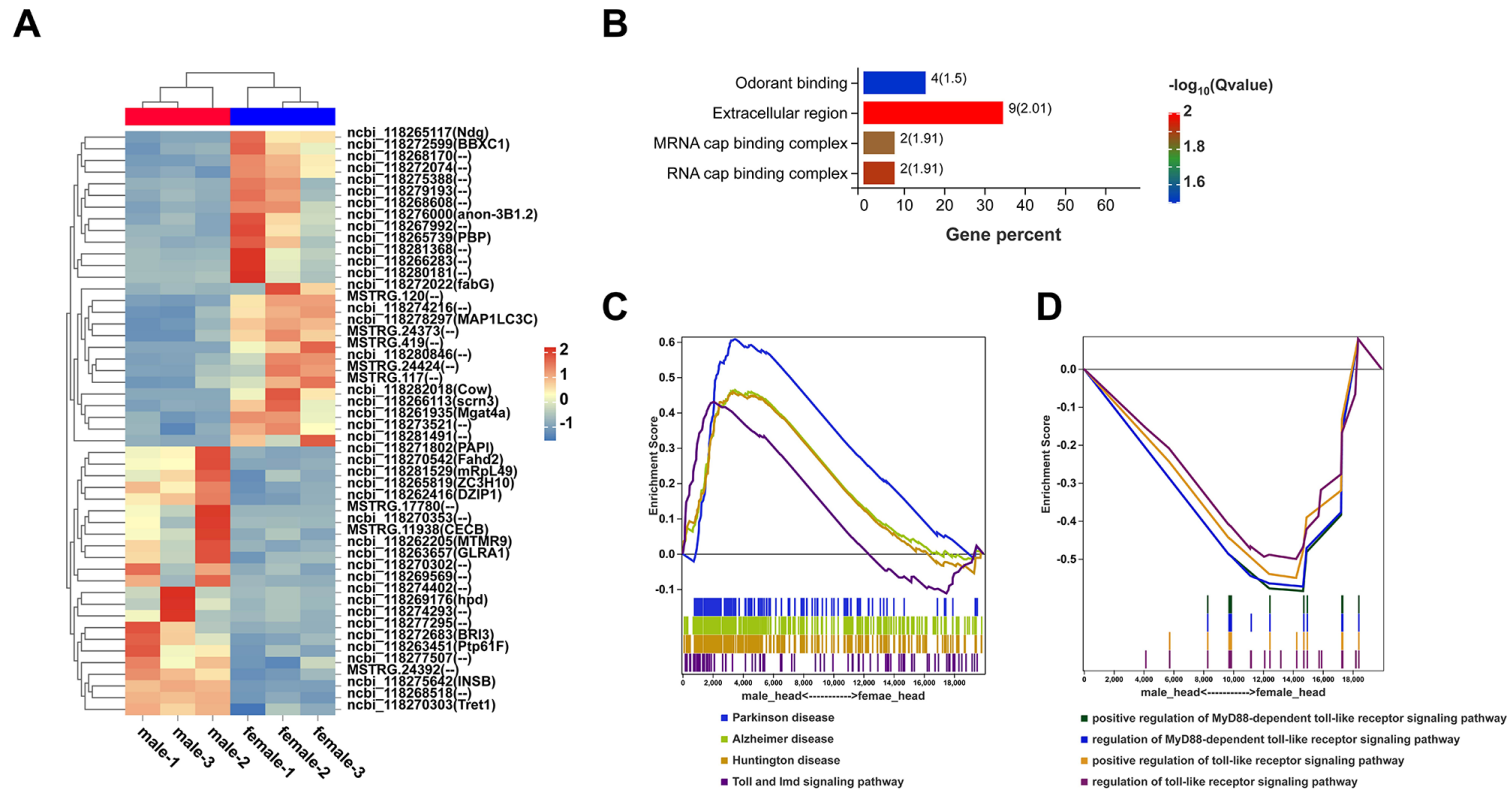
Differences in gene expression between female and male adult heads of *S. frugiperda* under conventional feeding. (**A**) Heatmaps of the DEGs between the female and male adult heads (FDR < 0.05 and |log2FoldChange|>1). (**B**) GO enrichment analysis of DEGs (*Q* value < 0.05). (**C-D**) Immune and neurodegenerative disease pathways of GSEA-KEGG and GSEA-GO (|NES|>1, FDR < 0.25).

To further investigate whether there was sexual dimorphism in the female and male heads of *S. frugiperda*, a comparative transcriptomic analysis of 3-day-old female and male adult heads was performed. There were 34 differentially expressed genes (DEGs) (|log2-fold change|>2, false discovery rate [FDR] < 0.05, Table S3) identified, of which 12 DEGs were upregulated and 22 DEGs were downregulated in the comparison group of conventional male heads and female heads (Fig. 3A). Furthermore, GO enrichment analysis indicated that the DEGs were mainly enriched in odorant binding, extracellular region, and mRNA cap binding complex (Fig. 3B). Among them, four genes encoding odorant-binding proteins (OBPs) (ncbi_118265739, ncbi_118267992, ncbi_118275388, ncbi_118279193) exhibited significantly reduced expression in the male head (Fig. 3B). Genes related to the RNA cap binding complex were also decreased in male heads (Fig. 3B). This implies that male adults may be more responsive to the environment than female adults.

To further explore the potential biological processes associated with immune response activity, we used gene set enrichment analysis (GSEA). The results of GSEA-KEGG showed that gene sets of neurodegenerative diseases and the Toll and Imd signaling pathways were more significantly upregulated in male adults than in female adults (Fig. 3C), whereas Toll-like receptor (TLR) signaling gene sets were enriched in female adults through GSEA-GO analysis (Fig. 3D). These data indicate that male and female adults perform immune activity in sexually distinct patterns.

### Physiological response of the adult head to gut dysbacteriosis

**Fig. 4:**
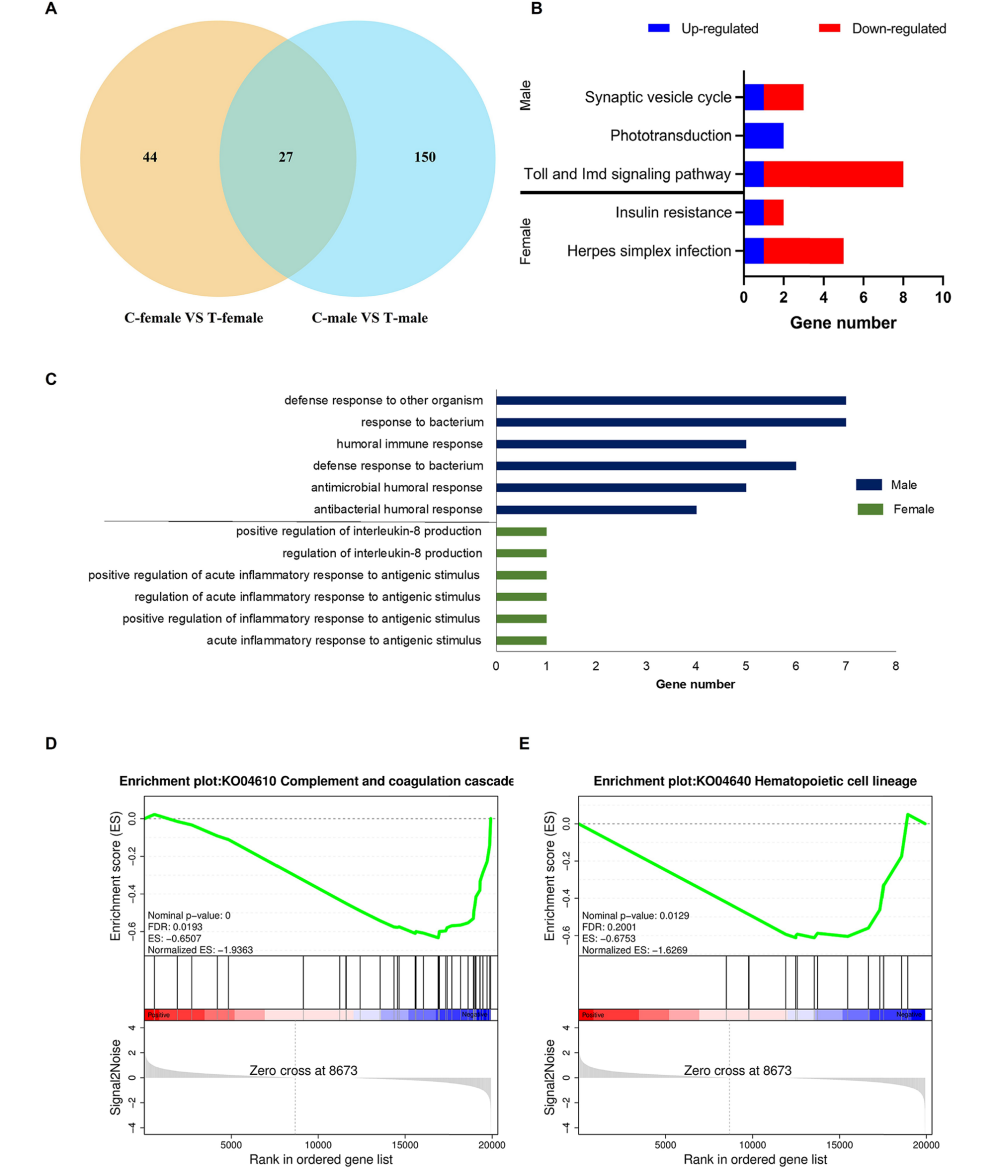
GO and KEGG analysis of differentially expressed genes in the adult head of *S. frugiperda* after gut dysbacteriosis. (**A**) Venn diagram of differentially expressed genes between control adult heads and antibiotic-treated adult heads. (**B**) KEGG enrichment analysis of DEGs in the heads of female and male adults. (**C**) GO enrichment analysis of the downregulated DEGs in the heads of female and male adults. (**D-E**) Dataset showing that two immune-related pathways were downregulated in the heads of female adults with gut dysbacteriosis as analyzed by GSEA-KEGG enrichment (NES|>1, FDR < 0.25).

To explore whether the gut microbiota influences gene expression in the *S. frugiperda* adult head, transcriptomic analysis of female and male adult heads was performed. Gut dysbacteriosis resulted in 20 upregulated and 27 downregulated genes in female adult heads (Table S4), and 40 upregulated and 44 downregulated genes in male adult heads (Fig. S1; Table S5). Additionally, the data revealed that 27 DEGs in total were common in female and male adult heads, while 44 DEGs were unique in differentially expressed gene sets of female adult heads and 150 DEGs were unique in male groups (Fig. 4A). Interestingly, DEGs critical for brain and neural development, including the lactosylceramide 4-alpha-galactosyltransferase gene (*A4galt*), facilitated trehalose transporter Tret1 (*Tret1*), 5-methylcytosine rRNA methyltransferase nsun (*nsun4*), probable galactose-1-phosphate uridylyltransferase (*Galt*), MICOS complex subunit Mic60 (*Mitofilin*), solute carrier family 2-facilitated glucose transporter member 1-like (*SLC2A3*), granzyme-like protein 1 (*snk*) and gamma-aminobutyric acid receptor subunit delta (*GABRB3*), were significantly downregulated in treated female adult heads. However, only two genes encoding the major heat shock 70 kDa protein Ba (*Hsp68*) and sialin (*SLC17A5*) essential for brain development and immunity, were significantly upregulated in treated female heads. Moreover, the mRNA levels of genes including *Tret1*, *Mitofilin*, *SLC2A3* and *GABRB3* were also inhibited in treated male heads, and there were two unique downregulated genes in male adult heads: octopamine receptor Oamb (*Oamb*) and sodium- and chloride-dependent GABA transporter 1 (*SLC6A1*) (Table S4-S5). These data showed that gut dysbacteriosis might also affect the head nervous system of *S. frugiperda*.

For female heads, KEGG enrichment demonstrated that the most affected pathway was linked to the interferon (IFNα/β) response within the herpes simplex infection pathway (*p* < 0.05), whereas treated male adult heads showed a closer association with the Toll and Imd signaling pathways (*p* < 0.05) (Fig. 4B). In addition, GO enrichment analysis indicated that a few DEGs from male heads were involved in biological processes of the humoral immune system, but the DEGs in female samples were concentrated in the inflammatory response of biological processes (*p* < 0.05) (Fig. 4C). Furthermore, two pathways in the immune system were enriched in control female adult heads according to GSEA-KEGG analysis: complement and coagulation cascades and hematopoietic cell lineage (Fig. 4D-E). Altogether, these results suggest that gut dysbacteriosis has significant and sex-specific impacts on the head transcriptome of *S. frugiperda* male and female adults, especially in the immune system.

### Gut dysbacteriosis diminishes the head immune response

**Fig. 5:**
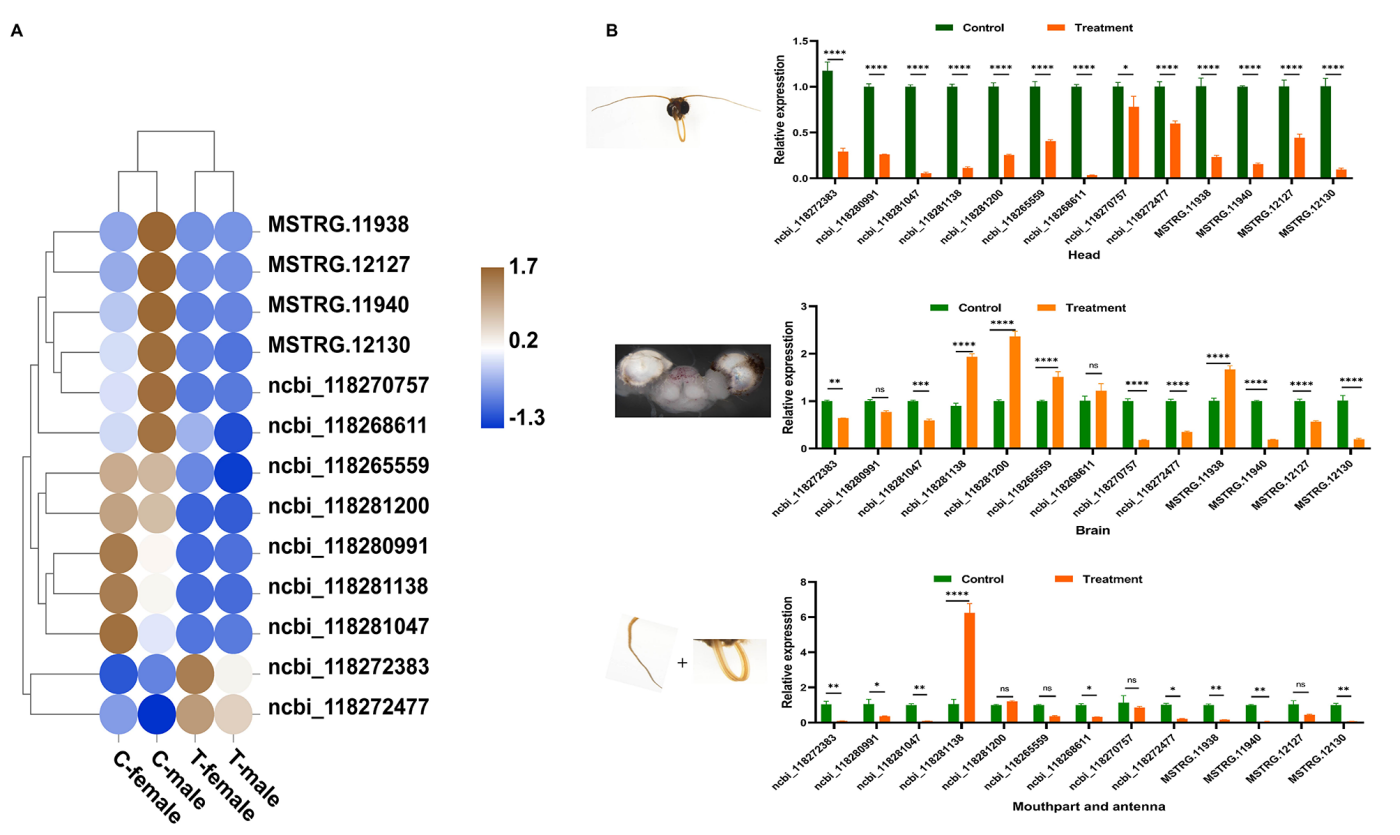
Expression levels of network genes in adults with gut dysbacteriosis and control adults of *S. frugiperda*. (**A**) Heatmap showing the changes in the expression of the key genes and their functional pathways. (**B-B**”) qRT‒PCR analysis of the mRNA expression of genes involved in the immune pathways in the female and male head (**B**), brain (**B**’) and antenna (B”) between control and gut dysbacteriosis adults. The data are presented as the means ± SM (n = 3). The significance of the differences between the treatment and control was statistically analyzed by using Student’s *t* test at *p* < 0.05 (*), *p* < 0.01 (**), *p* < 0.001 (***), and *p* < 0.0001 (***)

To further identify downregulated immune effectors after gut dysbacteriosis, the expression patterns of DEGs related to immunity in the adult heads, brain and antennae were analyzed. The results showed that the mRNA levels of coding zinc finger antiviral protein (ZAP) genes, including *HIVEP2* (ncbi_118280991), *GDNF-inducible zinc finger protein 1-like* (ncbi_118281138), *zinc finger protein 99-like* (ncbi_118281047) and *ZNF131* (ncbi_118281200) in the interferon pathway, was downregulated in treated female heads (Fig. 5A), while mRNA levels of *HIVEP2* and *GDNF-inducible zinc finger protein 1-like* also showed significant decrease upon dysbacteriosis in male adult heads but *zinc finger protein 99-like* and *ZNF131* had no differences (Table S6; Fig. S2). The mRNA levels of *serine/threonine protein kinase Pelle* (ncbi_118270757), *peptidoglycan-sensing receptor* (*PGRP*, ncbi_118268611), *cecropin A* (*CecA*, MSTRG.11940; MSTRG.12130) and *cecropin B* (*cecropin B*, MSTRG.11938; MSTRG.12127) in the Toll and Imd signaling pathways were drastically reduced in treated male adult heads (Fig. 5A), whereas all of these genes had no significant differential expression in female adult with dysbacteriosis (Table S7; Fig. S3). The expression of these immune response-related DEGs was confirmed by qRT-PCR analysis (Fig. 5B-B”). In brief, gut dysbacteriosis results in remarkable expression differences in immune genes in the *S. frugiperda* head in a sex-manner.

## Discussion

### Antibiotic-induced gut microbiota perturbation

The α-diversity and LEfSe analyses revealed dramatic alterations in the gut microbiota composition and diversity under conditions of antibiotic exposure (Fig. 1C-D). The abundances of the main species within the phylum Firmicutes and the genera *Enterococcus* and *Enterobacter* were significantly decreased in treated adult guts (Fig. 1A-B). Firmicutes have been reported to play key roles in nutritional supplementation, energy absorption and host immunity [58]. Moreover, *Enterococcus* and *Enterobacter* contribute to the synthesis of vitamins and pheromones, the degradation of plant compounds and nitrogen fixation [59, 60]. Additionally, the PTS system is used for bacteria to absorb carbohydrates and their derivatives through the phosphorylation cascade into the cell [61], and the bacterial PTS was remarkably repressed (Fig. 1E-F). The existence of gut-dominant microorganisms is one of the most important reasons that *S. frugiperda* are able to feed on various plants and have strong adaptability and resistance. LEfSe analysis revealed that the genera *Enterococcus* and the family Lactobacillales have lower abundance in the antibiotic-treated groups, which are associated with serotonin, dopamine, acetylcholine, histamine and SCFAs [62]. Therefore, we speculated that the differences in metabolites caused by changes in microbial composition might affect immune cells by accessing the brain or around the brain, and *Enterococcus* might be a core bacterium that links the gut-brain relationship in *S. frugiperda.*

### Expression difference between untreated female heads and male heads

A comparison between male and female adult heads revealed only 50 differentially expressed genes (Fig. 3A). In contrast, we found that the expression of four key coding genes of OBPs was greater in female adult heads (Fig. 3A-B). Insect chemosensory systems play important roles in reproductive success and survival, while OBPs are essential for detecting and distinguishing specific odors by transporting odorant molecules to olfactory receptors [63]. In the natural environment, the olfaction, gustation, vision and tactile sensing systems of female adult insects are simultaneously used to recognize suitable egg-laying locations and reduce competitive pressure [64]. Furthermore, DEGs of the RNA cap binding complex and the Toll-like receptor signaling pathways associated with inflammatory factors all showed higher expression in female heads (Fig. 3B and D). TLRs play significant roles in the host innate immune system, which can recognize various classes of pathogens, including bacteria, fungi, viruses and protozoa [65], and TLRs are necessary for immune cell survival, regulation and proliferation [66]. These data suggest that female *S. frugiperda* adults have a stable immune system, which helps female adults to better resist external environmental invasion for reproduction and egg laying. However, male adults had higher expression within the Toll and Imd signaling pathways and a different humoral immune response to combat microbial infection (Fig. 3C). Male and female innate immune responses are different. For example, the neuronal diseases Parkinson’s disease [67] and Alzheimer’s disease [68] show sex differences. Additionally, gender differences in brain immune function have also been reported across diverse species, with females generally having more dynamic immune function than males [69]. The Toll­like receptor 7 (TLR7) gene is located on the X chromosome and can escape X inactivation. Therefore, the expression level of TLR7 in females is higher than that in males [70]. Interferon­α production in cells from women is higher than that in cells from men when peripheral blood mononuclear cells (PBMCs) are exposed to TLR7 ligands in vitro [71]. Our results also indicate that the Toll­like receptor signaling pathway shows higher expression in the female adult heads (Fig. 3D), which is consistent with reports in mammals. The reasons for this biological phenomenon might be genetic mediators, age and reproductive status, hormonal mediators and environmental mediators [72].

### Female and male heads respond differently to gut dysbacteriosis

RNA-seq analysis of adult heads showed that gut dysbacteriosis altered the expression of more transcripts in males than that in females (Fig. 5A). Gut dysbacteriosis induced the transcriptional downregulation of antimicrobial peptides (AMPs) in the treated male *S. frugiperda* adult heads, and the repression of ZAP involved in the interferon IFNα/β pathway in the treated female *S. frugiperda* adult heads (Fig. 5). ZAP, as a kind of restriction factor, mediates the degradation of viral mRNA, inhibits viral translation and ultimately suppresses viral replication [73]. Thus, the gut microbiome likely contributes to the immune system in the head from *S. frugiperda* adults in a sex-specific manner. The gut microbiota affects the proportions, migration and functions of various immune cells [74]. Numerous examples have illustrated that gut bacteria can control both innate and adaptive immune responses on the mucosal surface during infection and inflammation [75]. However, how gut microbes control microglial maturation and innate immune reactions in the insect head remains incompletely understood. Importantly, both adult male and female heads of gut dysbacteriosis showed marked decreases in the expression of genes linked to brain and neural development compared with the levels in conventionally raised *S. frugiperda* (Table S4-S5). Neurotransmitters that transmit information between neurons are essential for brain functions and behaviors. Recent studies have indicated that microorganisms regulate the stimulation of glial cells by producing microbial metabolites. For example, SCFAs, neurotransmitters and gut hormones [76, 77] enter the circulatory system and signal to the host brain in this way [78]. Some studies have also found a cross-regulatory mechanism between the gut nervous system and the immune system. This once again confirms that gut microbiota can affect the brain through the immune system [79]. For example, compared to conventional flies, *Drosophila* are hyperactive under axenic conditions [80]. Additionally, the results of evaluating gene expression differences through qRT‒PCR indicated that DEGs related to the immune pathway changed more significantly in the brain than in other tissues (Fig. 5B-B”). According to reports, the toll channel in the central nervous system promotes immune responses by providing key factors, but excessive immune responses can cause damage to neurons [81]. Our study showed that adult brains of *S. frugiperda* are particularly affected when complex microorganisms are lacking. Sex-specific differences reveal that male adults are more sensitive to changes in microbial communities than females. We speculate that the immune signaling proteins of female adults are of the stable and constitutive type, while the immune proteins of male adults are of the susceptible and inducible type. Furthermore, the differences in microbial composition between female adults and male adults resulted in different regulatory functions of metabolic metabolites. More research is needed to confirm these findings and better understand sex, microbiota manipulation, microbiota-linked metabolites, and immune responses. Therefore, we will further explain the relationship between gut bacteria and the brain by supplementing the microbial community and conducting a comprehensive analysis. We will also detect behavioral changes in the treated *S. frugiperda*.

## Conclusion

In summary, we demonstrate the head transcriptomic differences between female and male adults of *S. frugiperda*. In particular, we found the potential impact of gut bacteria on the head immune pathway. Gut microbiota dysbiosis may drive inhibition of the IFN pathway in female adult heads, but Toll and Imd signaling pathway in male adult heads, thereby decreasing the expression level of genes encoding antibacterial protein. Gut microbiota dysbiosis also resulted in the gene expression changes involved in the neurodevelopment. Furthermore, *S. frugiperda* adults showed sex-specific response differences to gut dysbiosis. Further research involved in the underlying mechanism by how gut bacteria affect head immune pathways and brain neurodevelopment need to be substantiated. In brief, our study suggests a probable linkage between gut microbiota dysbiosis and the brain immunity in *S. frugiperda*. These findings benefit our understanding of the impact of gut symbionts on the ecology and evolution of insects and provide some model references for further research on psychiatric disorders.

### Electronic supplementary material

Below is the link to the electronic supplementary material.

Supplementary Material 1

Supplementary Material 2

Supplementary Material 3

Supplementary Material 4

## Data Availability

All data needed to evaluate the conclusions in this paper are presented in the paper and the Supplementary Materials. All data of the raw reads have been deposited into the NCBI Sequence Read Archive (SRA) database (accession numbers PRJNA986514, PRJNA986662 and PRJNA983288). The datasets generated and/or analyzed during the current study are available from the corresponding author on reasonable request.
