# Supplementary figures and images for: Gut dysbacteriosis induces expression differences in the adult head transcriptome of Spodoptera frugiperda in a sex-specific manner
Source: BMC Microbiol. 2023 Dec 7;23:388. doi: 10.1186/s12866-023-03089-0 (PMC10702092; doi:10.1186/s12866-023-03089-0)

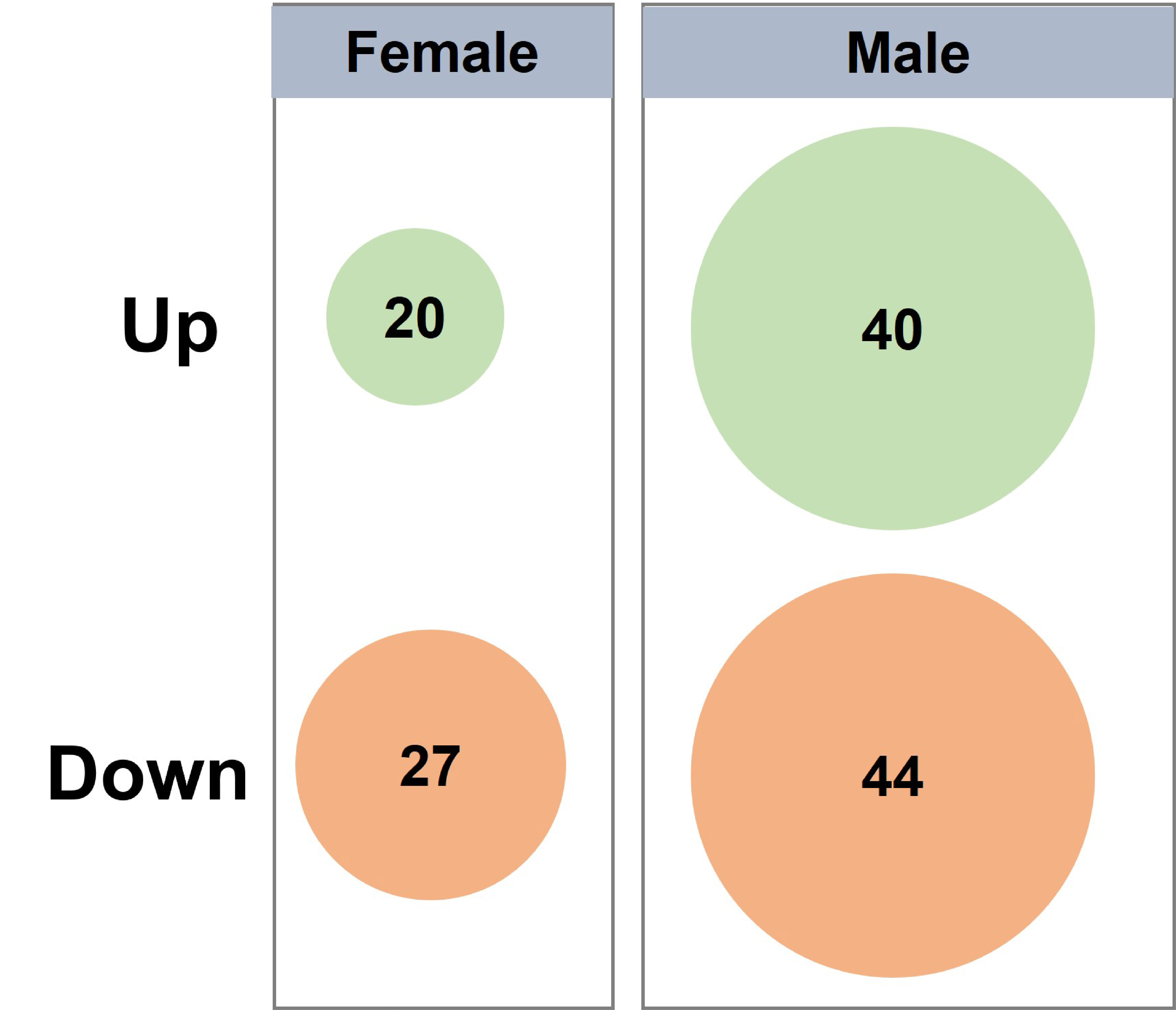

Supplement: Supplementary file 2 — Supplementary Material 2 [file 12866_2023_3089_MOESM2_ESM.jpg]

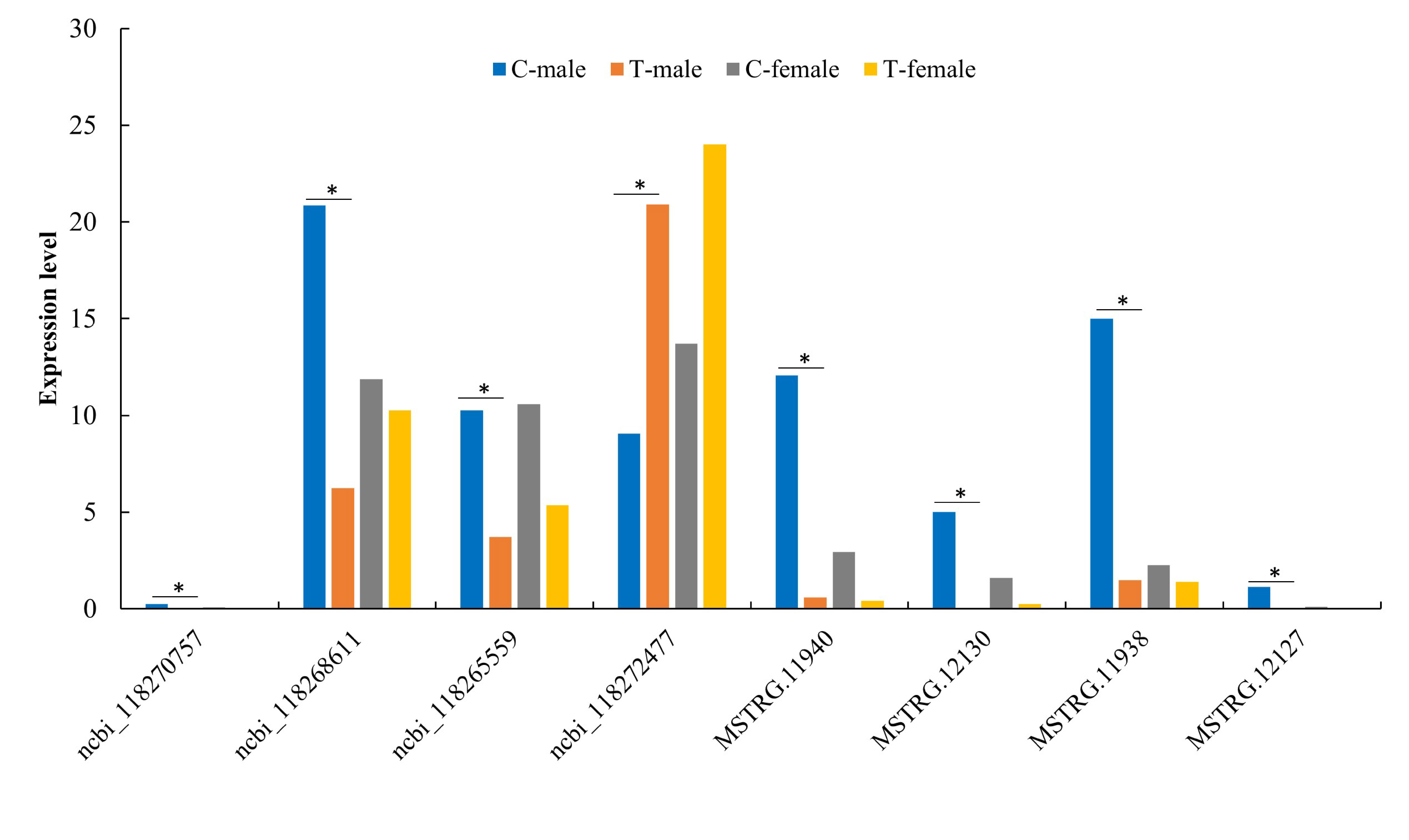

Supplement: Supplementary file 3 — Supplementary Material 3 [file 12866_2023_3089_MOESM3_ESM.jpg]

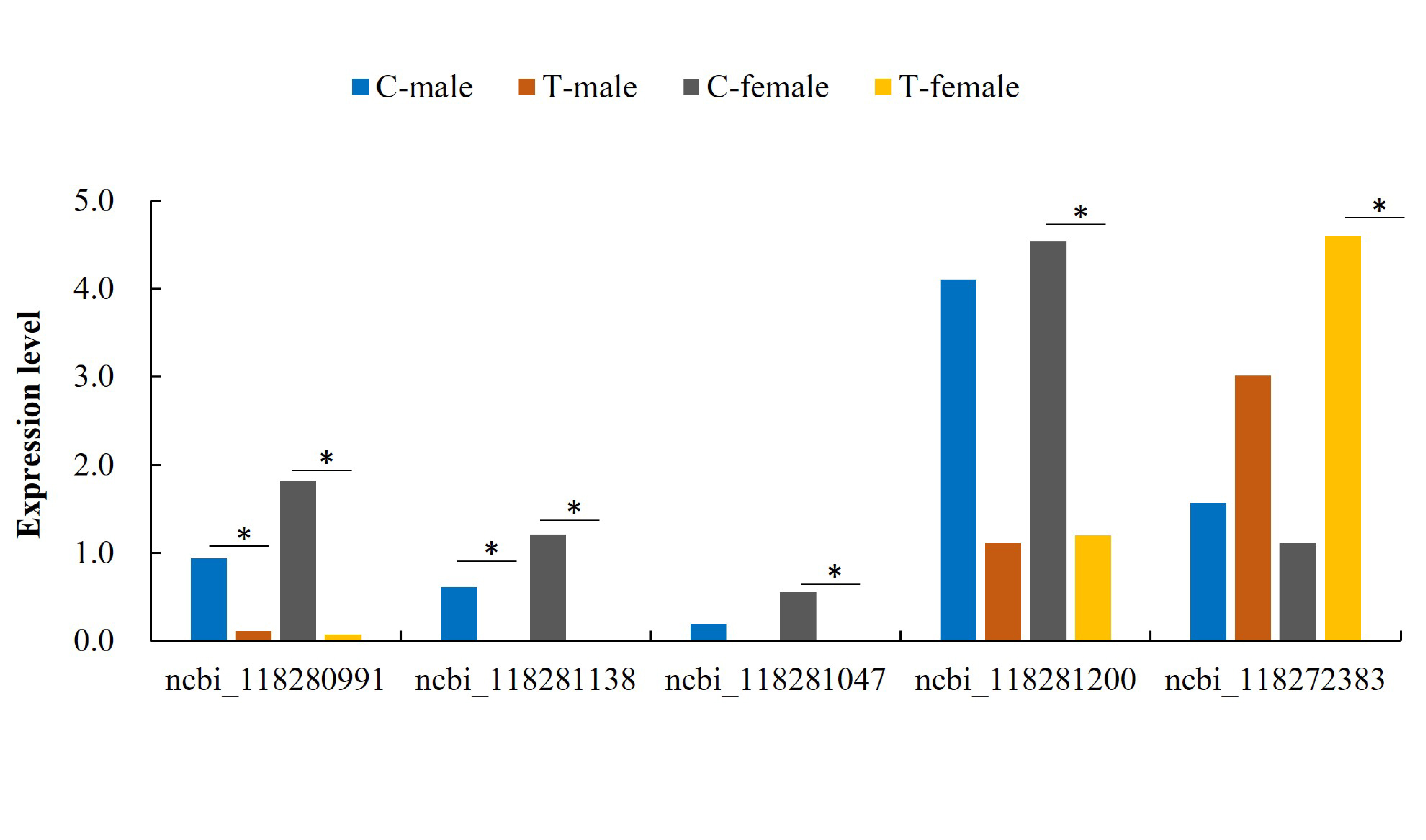

Supplement: Supplementary file 4 — Supplementary Material 4 [file 12866_2023_3089_MOESM4_ESM.jpg]
